# Development and validation of a metamemory maturity questionnaire in the context of English as a foreign language

**DOI:** 10.1186/s40468-021-00141-6

**Published:** 2021-10-01

**Authors:** Payam Nour, Rajab Esfandiari, Abbas Ali Zarei

**Affiliations:** Department of English Language, Faculty of Humanities, Imam Khomeini International University, P.O. Box 34149116818, Qazvin, Iran

**Keywords:** Attentiveness, Maturity, Metamemory, Questionnaire, SEM, Strategy

## Abstract

To determine the inherent components of language learners’ capacity for metamemory maturity, the researchers drafted a metamemory maturity (MMM) questionnaire based on Hultsch et al.’s (Memory self-knowledge and self-efficacy in the aged, Springer-Verlag 65–92, 1988) model. The volunteer participants were a heterogeneous sample of 356 male and female English as a Foreign Language (EFL) teachers and student teachers with various age ranges, teaching experiences, and educational backgrounds. Through a series of factor analytic procedures and structural equation modeling, the final draft of the questionnaire with 30 binary Likert-scale items was validated. Statistics confirmed acceptable measures of internal consistency as well as convergent and discriminant validity. The newly designed MMM questionnaire consisted of three main components of memory strategy use (12 items), memory attentiveness (6 items), memory factual awareness (6 items), and a moderator component of confidence and affect (6 items). The researchers highlight the implications of this questionnaire to provide the teachers with an instrument to analyze the needs of EFL learners for metamemory enhancement strategies.

## Introduction

A great deal of any learning process concerns the recollection of to-be-recalled pieces of information (Elimam & Chilton, 2018; Logan et al., 2012). Thus, learning a second/foreign language is drastically dependent on how efficiently language learners’ memory system is used and manipulated (Durand López, 2021; Rakow et al., 2010; Rankin, 2017). Memory capacity on its own and without enhancement strategies will become static or even deteriorated (Dunning & Holmes, 2014; Gathercole et al., 2019; Strobach & Schubert, 2016). In other words, memory operations tend to be limited and inefficient if the memory system remains untrained. Hence, reasonable manipulation of memory systems would cease the loss (Dunlosky & Metcalfe, 2009), because memory enhancement strategies pave the way for influencing the neurology of the brain and boosting the retrieval of stored knowledge (Baddeley et al., 2015; Li et al., 2019; Salmi et al., 2018). Therefore, language learners tend to pursue effective ways to uplift the productive capacity of memory by closely observing, re-evaluating, and adopting necessary regulations of memory functionality (Dunlosky & Thiede, 2013).

From epistemological and psychological perspectives, the Greek prefix “meta” is usually attached to a word to denote a discussion *about* that concept or process (Hertzog & Curley, 2018). In the same vein, Martinez (2006) defined metacognition as over-thinking, monitoring, and controlling one’s thoughts. To elaborate the breadth of metacognitive functioning, Martinez proposed a taxonomy of three main categories for metacognition, namely, metamemory, meta-comprehension, and problem-solving and logical thinking. Metamemory is roughly known as enhancing self-awareness, regulating one’s own memory processes, constructing knowledge, generating awareness, and self-monitoring of memory functions (Dunlosky & Thiede, 2013; Hertzog & Curley, 2018). Therefore, metamemory is just one subcomponent of metacognition, which influences higher-order thinking, and learning, in a variety of ways, especially in terms of making effective use of limited cognitive resources, using strategies, and tracking comprehension (Bjork & Bjork, 2011; Dunlosky & Thiede, 2013; Stone, 2000).

Metamemory in theory and practice has recently been scrutinized in a number of research studies (e.g., Blake et al., 2015; Cottini et al., 2018; Dunlosky & Bjork, 2008; Dunlosky & Metcalfe, 2009; Dunlosky & Thiede, 2013; Einstein & Mcdaniel, 2007; Maki et al., 2009). Metamemory has been studied from different standpoints such as intrinsic and extrinsic metamemory typology (Susser & Mulligan, 2019), the effect of expectancy illusion on metamemory (Schaper & Bayen, 2021), the effect of mnemonic devices on metamemory (Mieth et al., 2021), different strategy choices for enhancing metamemory (Park et al., 2018), and its relationship with the cognitive offloading (Hu et al., 2019). However, experimental studies have mainly focused on the relevant courses of action in clinical psychology and ordinary life habits to measure the clients’ metamemory construct in the course of tasks and activities (Dixon & Hultsch, 1983; Tonković & Vranić, 2011; Troyer et al., 2019; Van Ede & Coetzee, 1996).

In a number of studies, SLA researchers have recently shown their interest in encouraging language learners’ manipulation of metacognitive mechanisms (Bui & Kong, 2019; Cer, 2019; Han, 2020; Zhang & Zhang, 2019). However, a marginal number of works have been directed particularly to the substantial role of metamemory maturity in the acquisition process of a second/foreign (L2) language. By definition, *maturity* entails language learners’ constant evolvement of responsibility, knowledge, reflectiveness, self-esteem, autonomy, and cognizance in a certain task (Dermanova & Manukyan, 2010). Undoubtedly, prior to any metamemory manipulation, an observation of L2 learners’ metamemory maturity is required. Thus, developing an instrument to measure the gradual improvement in the status quo of metamemory seems necessary, since the existing questionnaires have no valid applications to language learners in L2 contexts.

Some well-known metamemory questionnaires are the Metamemory in Adulthood (MIA) (Dixon & Hultsch, 1983); the Metamemory, Memory Strategy, and Study Technique Inventory (MMSSTI) (Van Ede & Coetzee, 1996); the Everyday Memory Questionnaire (EMQ) (Royle & Lincoln, 2008); and the Self-Evaluation of Memory Systems Questionnaire (SMSQ) (Tonković & Vranić, 2011). The questionnaires have commonly adopted a neurocognitive approach to the nature of metamemory, which are mostly rooted in how clinical patients verbalize and picture their own memory processes. Despite reportedly satisfactory psychometric properties, they are disputed for such critical shortcomings as a large number of items as a cause for boredom and distraction to the respondents, disarrangement of the items, unexamined convergent and discriminant validity measures, or low generalizability. Widely used in psychotherapeutic contexts, these questionnaires can hardly account for the dynamic psychological attributes in language learners. Hence, the absence of suitable instruments for measuring metamemory maturity in other research domains such as L2 language teaching/learning seems inevitable. To fill the void, the researchers in the present study conceptualized the components of metamemory to measure its maturity, with a specific focus on the EFL learners and student teachers. The drafted and validated questionnaire in this study was labeled as the *metamemory maturity* (MMM) questionnaire.

The incentive behind developing the MMM questionnaire was twofold. First, due to the dynamic nature of second/foreign language learning and teaching context in which the various interacting factors have serious impacts on both quality and quantity of language learning, the acting variables are seemingly different from those in therapeutic and clinical settings. Therefore, the sampling errors caused by such variations would lead to fluctuating patterns of data in non-clinical educational (i.e., EFL) contexts which eventually deteriorate the reliability of the results if the available questionnaires are applied (Best & Kahn, 2006). In order to reduce the margin of errors, the theoretical framework of the MMM questionnaire was specifically grounded on the target population of EFL teachers and EFL student teachers. Secondly, the MMM questionnaire was constructed on solid theoretical and statistical grounds to compensate for the shortcomings in other questionnaires, such as large number of items, small sample size, and subsequent low generalizability index, as well as fallacies in discriminating components of metamemory construct. The items in the MMM questionnaire are based on the well-known model of metamemory introduced by Hultsch et al. (1988) with four main components of *memory factual knowledge*, *memory monitoring*, *memory self-efficacy*, and *memory-related affect*. In their original and comprehensive model of metamemory, Hultsch et al. elaborated on these components in detail.

The first component of metamemory in Hultsch et al. (1988), *memory factual knowledge*, is defined as someone’s knowledge of what memory is and what pertinent tasks and strategies can be used for better results in a memory-demanding situation (Dunlosky & Thiede, 2013; Dunning & Holmes, 2014; Gathercole et al., 2019; Strobach & Schubert, 2016). Memory factual knowledge encompasses a wide range of principal and practical undertakings (Hultsch et al., 1988). Some language learners appear incognizant of how their memory works and where the plans for storing, processing, and retrieving language input are grounded (Robinson, 2017; Spanoudis & Demetriou, 2020). Incorporating their memory factual knowledge, researchers maintain vigilance to employ memory enhancement strategies (Kazi et al., 2019). General knowledge of diets, hydration, bedtime, and sleep deprivation effects, as well as sufficient knowledge of how memory operates, is one of the instances of memory factual knowledge in metamemory (Cousins & Fernández, 2019; Peng et al., 2020; Tamminen et al., 2020).

The second component of metamemory, *memory monitoring*, refers to someone’s close observation of self-memory use in memory-demanding tasks (Hultsch et al., 1988). In memory monitoring, the process of applying memory factual knowledge to memory tasks is keenly followed (Huff & Bodner, 2013). Time allocation strategies (Ariel et al., 2009; Double & Birney, 2019; Tauber & Rhodes, 2012), spaced practice, re-studying, and scheduling (Carvalho & Goldstone, 2021; Kelley & Whatson, 2013; Logan et al., 2012; Son, 2010), as well as benefiting judgments of learning data (JOLs) (Janes et al., 2018; Myers et al., 2020), are some examples of memory monitoring.

*Memory self-efficacy* is the third component in Hultsch et al.’s (1988) model, which probes the extent the learners feel content about their own memory capacity and memory functionality. Aging, which is typically tinged with declines in memory potentiality, and lack of daily brain activity, which causes stagnancy of the memory systems, are reported as contributing factors to low satisfaction over memory efficacy (Bubbico et al., 2019; Li et al., 2019; Pfenninger & Singleton, 2019). Health issues also contribute to growing memory loss and subsequent dissatisfaction (Mandolesi et al., 2018). On the other hand, some factors such as education, effortful strategy use (Laine et al., 2018; Peng & Fuchs, 2017), and confidence-raising workout are popular remedies for low memory self-efficacy (Auslander et al., 2017; Boldt & Gilbert, 2019).

Finally, *memory-related affect*, as the fourth component, embeds the emotional factors playing roles in memory-demanding activities (Hultsch et al., 1988). Emotions by nature can either facilitate memory functions or cause cognitive impairments. Language learners with high anxiety level, for instance, are more vulnerable to memory loss (Riegel et al., 2015), or depressive EFL learners are reported as incompetent in those memory adaptive behaviors that result in effective language learning uptake (Staniloiu & Markowitsch, 2019).

As stated earlier, the term maturity encompasses a steady development in responsibility, knowledge, reflectiveness, self-esteem, autonomy, and cognizance in a certain task. In essence, a growing maturity in metamemory and metacognitive pursuits seems to be essential to the performance of those who are actively involved in the context of language learning and even teaching (Dunlosky & Thiede, 2013). Metamemory maturity is likely to improve the functionality, self-satisfaction, and awareness of language learners after a period of training (Baddeley et al., 2015). Therefore, it seemed critical to develop a valid and reliable instrument for identifying and measuring the components of metamemory maturity in the L2 context and to statistically confirm the soundness of its underlying components.

Reportedly, impairments in different stages of memory are bound to learning failure in general (Chein & Morrison, 2010; Daneman & Hannon, 2007; Oberauer et al., 2008) and language learning in particular (Carroll, 2004). In practice, countless language learners are blamed for their inefficient memory in retaining new words or language structures (Baddeley, 2003), and such infirmity can be easily conquered by training them to gain maturity in monitoring and manipulating memory use (Baddeley et al., 2015). Such training needs an instrument to obtain a vivid picture of the language learners’ memory status quo. However, the SLA community lacks a sound and comprehensive scale. To fill the gap, the researchers attempted to develop and validate a metamemory maturity questionnaire to address the target population of EFL language learners and teachers. The following research questions were raised and explored in this study:

- RQ1: What are the psychometric properties of the metamemory maturity (MMM) questionnaire in an EFL context?
- RQ2: What are the underlying components of the metamemory maturity (MMM) questionnaire?
- RQ3: To what extent does the structural model of metamemory (MMM) questionnaire fit the hypothetical model generated by relevant literature review?

## Method

### Participants

A total number of 356 participants voluntarily took part in the present study. They were selected through a snowball non-random sampling procedure (Heckathorn, 2002) from a pool of experienced EFL teachers and EFL student teachers at three private language institutes as well as student teachers in three universities in Iran. Table 1 summarizes the demographic information of the participants in this study.

**Table 1:** Demographic information of the participants in the study

Characteristics		Frequency	Percentage
Educational status	Diploma	100	28.1
	BA	171	48.3
	MA	76	21.1
	PhD	7	1.94
	Post-doctoral	2	0.56
Years of teaching experience	None	170	48
	1–3	88	24.7
	4–7	36	10.4
	8–10	16	4.5
	> 10	46	12.4
Age range	15–20	50	14
	21–30	191	53.7
	31–40	75	21.1
	> 40	40	12.2
Gender	Male	131	37
	Female	225	63

Since determining the sample size in this study was a major issue for running the statistical tests of exploratory and confirmatory factor analyses (EFA and CFA) as well as structural equation modeling (SEM), a widely noted approach to sample size estimation by Kline (2011) was adopted. Kline argued that to determine the optimal number of respondents to a questionnaire in the piloting phase, a sample size of 30 to 460 is required when the number of components in the conceptual model is three to eight. Because the MMM questionnaire was developed based on the four components of (1) memory factual knowledge, (2) memory monitoring, (3) self-efficacy, and (4) memory-related affect in Hultsch et al.’s (1988) model of metamemory, a minimum sample size of 360 participants was required.

Determining an appropriate sample size is a critical issue in SEM studies, but, unfortunately, an exact, agreed-upon consensus does not exist in the literature (see Wang & Wang, 2020). There is no absolute standard concerning an adequate sample size and no rule of thumb that applies to all SEM contexts (see Wang & Rhemtulla, 2021 for an update). The determination of sample size, as Wang and Wang (2020) neatly summarized, depends on a large number of factors, including the number of free parameters and the number of indicators per latent variable, data characteristics, and the model being tested, such as reliability of the observed indicators, study design (e.g., cross-sectional versus longitudinal), degree of data multivariate normality, handling of missing data, model complexity, and the model estimators. Given the multiplicity of factors in the determination of the SEM sample sizes, researchers conducting SEM studies resort to rules of thumb recommending either absolute minimum sample sizes (e.g., *n* = 100 or 200; Boomsma, 1985) or sample sizes based on model complexity (e.g., *n* = 5–10 per estimated parameter, according to Bentler & Chou, 1987; *n* = 3–6 per variable, according to Lee and Song, 2004). However, as Wang and Rhemtulla (2021) remind us, these rules of thumb “do not always agree with each other, have little empirical support … and generalize to only a small range of model types” (p. 1). By implication, the recommendations in the literature concerning SEM sample sizes either reflect theoretical orientations or are based on a very small number of empirical research studies.

Following model complexity to determine sample size in their SEM studies, researchers usually use the ratio of participates/cases to items/variables. Using even this approach, researchers appear to be divided over the minimum number of participants for a SEM analysis, so, while some researchers (e.g., Kline, 2016) consider five cases per variable to be the minimum sample size for a SEM study, some others like Lee and Song (2004) recommend the minimum number of three cases per variable for a SEM study. However, when the complexity model is used, researchers usually follow Kline’s recommendation for the minimum number of participants, as one of the anonymous reviewers has also pointed out. Therefore, in our study, following Lee and Song’s (2004) recommendation, we needed at least 216 participants to begin our study with, but according to Kline’s suggestion, a sample size of at least 360 participants was needed. The participants in the present study included 356 language teachers, which means four other language teachers had to complete the questionnaire. Although the absence of these very few participants may not generally affect the findings (mainly due to the robustness of the SEM test), we consider it to be a limitation of our study.

### Instrument formulation

For each of the components of Hultsch et al.’s (1988) model of metamemory (i.e., memory factual knowledge, memory monitoring, self-efficacy, and memory-related affect), a comprehensive review of the literature was conducted, the results of which were incorporated into a number of themes and operational definitions. These statements were later used to draft a total number of 80 Likert-scale items with twenty items allocated to each of Hultsch et al.’s (1988) metamemory components. In Table 2, the hypothetical components of the MMM questionnaire and their encoded themes are presented with some selected items in its first draft.

**Table 2:** Initial components and retrieved themes in the MMM questionnaire

Component	Theme	Example
Memory factual knowledge	Memory capacity Memory quality Memory of the past Memory strategies	Item 7. There are certain memories that I think I will never forget.
Memory monitoring	Self-observation Memory management Time management	Item 38. When I have time limitation, I skip memorizing some difficult items.
Memory-related affect	Personal emotions Color codes Time management	Item 52. When I am sad, I memorize negatively loaded information better.
Memory self-efficacy	Self-evaluation Progressiveness	Item 70. I regularly challenge myself with memorizing difficult items.

Tables 3, 4, 5, and 6 show the four example items in the questionnaire with their theoretical background and reference entries. Each item represents one component in the final draft of the questionnaire (i.e., memory factual knowledge, memory monitoring, memory self-efficacy, and memory-related affect). In order to avoid acquiescence bias (Dornyei & Taguchi, 2010), a binary Likert scale was implemented in order to safeguard the participants against ambivalence in responding to the items.

**Table 3:** Memory factual knowledge; exemplar item with the source and reference

Source	Reference
*1. I think my memory has a limited capacity.*	
It was traditionally assumed that working memory (WM) capacity is an immutable individual characteristic, but research at the beginning of the 2000s showed that the WM capacity of children and young adults could be increased by using computerized training that allows more extensive training (often more than 12 h).	Jaeggi, S. M., Buschkuehl, M., Jonides, J. & Perrig, W. J. (2008). Improving fluid intelligence with training on working memory. *Proceeding of the National Academy of Sciences*. 105, 6829–6833.

**Table 4:** Memory monitoring; exemplar item with the sources and references

Sources	References
*40. I memorize better when I am worried about the time limit.*	
The cognitive component has been implicated in the performance decrements seen in individuals with high test anxiety. Feelings of anxiety could be interpreted as either facilitative or debilitative. However, this framework is based on flawed empirical research and is not supported by evidence from mainstream psychology literature.	Mowbray, T. (2012). Working memory, test anxiety and effective interventions: A review. *Australian Educational and Developmental Psychologist. 29, 2,* 141-156. Polman, R. & Borkoles, E. (2011). The fallacy of directional anxiety. *International Journal of Sport Psychology. 42,* 303-306.

**Table 5:** Memory self-efficacy; exemplar item with the source and references

Source	References
*72. I believe I will never be good at memorizing difficult items*.	
However, decreases in memory performance are also partly due to age-related changes in motivational factors, including loss of interest in performing classic laboratory memory tasks, a decline in one’s sense of control over memory, and a lack of confidence in one’s ability to use memory effectively in memory-demanding situations.	Desrichard, O., & Köpetz, C. (2005). A threat in the elder: The impact of task-instructions, self-efficacy and performance expectations on memory performance in the elderly. *European Journal of Social Psychology*, *35*, 537–552. Hess, T. M. (2005). Memory and aging in context. *Psychological Bulletin, 131,* 383–406. doi 10.1037/0033-2909.131.3.383. Lachman, M. E., Neupert, S. D., & Agrigoroaei, S. (2011). The relevance of control beliefs for health and aging. In K. W. Schaie & S. L. Willis (Eds.), *Handbook of the psychology of aging* (7th ed., pp. 175–190). New York: Elsevier.

**Table 6:** Memory-related affect; exemplar item with the source and reference

Source	Reference
*50. When I am sad, I memorize negatively loaded items better.*	
In addition to executive dysfunctions, for example, cognitive impairments in depression have often been associated with further memory-related dysfunctions.	Matthews, K., Coghill, D., & Rhodes, S. (2008). Neuropsychological functioning in depressed adolescent girls. *Journal of Affective Disorders, 111,* 113-118. doi: 10.1016/j.jad.2008.02.003

The initial draft of the questionnaire was reviewed by five experts, including two professors of applied linguistics and three experienced EFL teachers for the first round of content validity and theoretical saturation. Eight items were found vague and inappropriate, and thus, they were excluded. The final draft of the questionnaire included 20 items for the component *memory factual knowledge* (items 1 to 20), 18 items for the component *memory monitoring* (items 21 to 38), 20 items for the component *memory-related affect* (items 39 to 59), and 15 items for the component *memory self-efficacy* (items 60 to 75). With 72 finalized items from the expert opinion validation process, the second (final) draft of the questionnaire (with 72 items) was administered to 356 student teachers selected from three universities and three private language schools over the course of 2 weeks using the snowball method of sampling. All 356 participants responded to all items of the questionnaire. Due to the COVID-19 pandemic, the questionnaire was constructed in the online Google Forms platform and distributed to the participants through their emails or personal IDs on social media. The collected set of data were subjected to exploratory and confirmatory factor analysis (EFA and CFA) to determine the construct validity of the questionnaire (Osborne et al., 2008). In addition, the structural equation modeling (SEM) was conducted in order to define the path orientation of the underlying components in the multifaceted metamemory maturity construct and their factor loadings.

## Results

### Results for 72 items of the questionnaire

#### Reliability measure

Prior to statistical analysis, the researchers measured the reliability of the data (Cronbach’s *α* = 0.865) which was interpreted as a high internal consistency index for 72 items of the questionnaire. The reliability statistics eradicated a notable change if any of the items were removed from the set after probing item total statistics. Therefore, all the items were sustained to undergo factor extraction analysis.

#### The application of exploratory factor analysis (EFA)

Although the newly designed MMM questionnaire was drafted to map Hultsch et al.’s (1988) model, an EFA was conducted in order to avoid any bias towards setting up a metamemory maturity construct. The analysis was run on an Oblimin rotation of the collected responses from all 356 participants. The sampling adequacy was examined by Kaiser-Meyer-Olkin (KMO) (Kaiser, 1970). The threshold value of KMO is expected to score over 0.60. The KMO statistics for the data displayed a KMO value equal to 0.69; therefore, the assumption of sampling adequacy was met. Likewise, the chi-square *p*-value for Bartlett’s test of sphericity showed a significant difference (*p* = 0.00, < 0.05) between the matrix in the data set and the identity matrix.

As setting a strong set of data is often recommended in the literature for conducting EFA, the commonality values are always critical. The communality cutoff value is reported to be above 0.30 (Field, 2013). In the collected data, the main body of communalities in the output ranged from 0.60 to 0.73, with a few exceptions around 0.52. The items with moderate communality values (*n* = 4) were excluded from the data in the following statistical analysis in order to maintain the maximum strength in the data.

#### Factor extraction and retention

After a parallel analysis (PA), the explored eigenvalues were compared to a set of uncorrelated eigenvalues produced by the Monte Carlo algorithm (Horn, 1965). Accordingly, all the observed eigenvalues in the EFA matrix surpassed the uncorrelated eigenvalues in the Monte Carlo algorithm, which warranted the appropriacy and acceptability of the observed eigenvalues (see Table 7).

**Table 7:** Factor extraction total variance explained

Total variance explained						
Component	Initial eigenvalues			Extraction sums of squared loadings		
	Total	% of variance	Cumulative %	Total	% of variance	Cumulative %
1	7.555	10.493	10.493	7.555	10.493	10.493
2	3.035	4.216	14.709	3.035	4.216	14.709
3	2.685	3.729	18.438	2.685	3.729	18.438
4	2.079	2.888	21.325	2.079	2.888	21.325
5	1.953	2.713	24.038	1.953	2.713	24.038
6	1.807	2.509	26.547	1.807	2.509	26.547
7	1.721	2.390	28.937	1.721	2.390	28.937
8	1.698	2.358	31.296	1.698	2.358	31.296
9	1.626	2.258	33.554	1.626	2.258	33.554
10	1.596	2.216	35.770	1.596	2.216	35.770
11	1.537	2.135	37.905	1.537	2.135	37.905
12	1.523	2.116	40.021	1.523	2.116	40.021
13	1.438	1.998	42.019	1.438	1.998	42.019
14	1.411	1.959	43.978	1.411	1.959	43.978
15	1.401	1.945	45.923	1.401	1.945	45.923
16	1.373	1.907	47.830	1.373	1.907	47.830
17	1.339	1.859	49.689	1.339	1.859	49.689
18	1.282	1.781	51.470	1.282	1.781	51.470
19	1.226	1.702	53.173	1.226	1.702	53.173
20	1.203	1.670	54.843	1.203	1.670	54.843
21	1.144	1.588	56.431	1.144	1.588	56.431
22	1.142	1.586	58.018	1.142	1.586	58.018
23	1.080	1.500	59.517	1.080	1.500	59.517
24	1.069	1.485	61.002	1.069	1.485	61.002
25	1.040	1.444	62.446	1.040	1.444	62.446
26	1.027	1.426	63.872	1.027	1.426	63.872
27	.987	1.371	65.243			
28	.985	1.368	66.611			
29	.934	1.297	67.908			
30	.915	1.271	69.179			
31	.891	1.237	70.416			
32	.882	1.225	71.641			
33	.859	1.193	72.834			
34	.839	1.166	74.000			
35	.814	1.131	75.131			
36	.792	1.100	76.231			
37	.777	1.079	77.310			
38	.765	1.063	78.372			
39	.735	1.021	79.393			
40	.723	1.005	80.398			
41	.707	.982	81.380			
42	.678	.941	82.321			
43	.677	.940	83.261			
44	.638	.886	84.147			
45	.628	.872	85.020			
46	.596	.827	85.847			
47	.592	.822	86.669			
48	.589	.818	87.487			
49	.556	.772	88.259			
50	.525	.729	88.989			
51	.506	.703	89.691			
52	.498	.692	90.383			
53	.496	.689	91.072			
54	.483	.671	91.742			
55	.464	.644	92.387			
56	.445	.618	93.005			
57	.424	.589	93.595			
58	.418	.581	94.176			
59	.401	.557	94.733			
60	.382	.531	95.264			
61	.351	.487	95.751			
62	.348	.484	96.235			
63	.340	.473	96.708			
64	.324	.450	97.157			
65	.315	.437	97.594			
66	.312	.433	98.027			
67	.276	.384	98.411			
68	.265	.368	98.779			
69	.257	.357	99.136			
70	.230	.319	99.455			
71	.215	.299	99.754			
72	.177	.246	100.000			

**Fig. 1:**
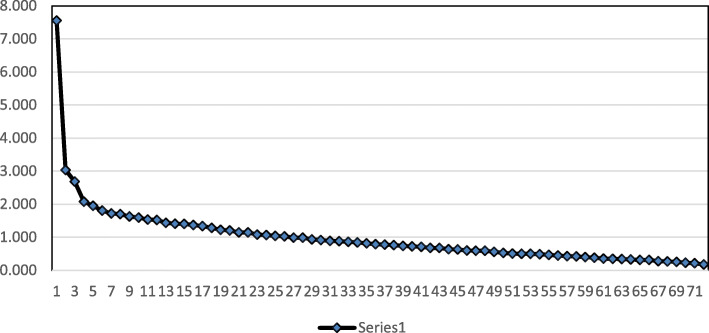
The distribution of the extracted factors

Twenty-six components were detected with eigenvalues above 1 (Kaiser’s criterion component, 1960) which outnumbered the components in the Hultsch et al. (1988) model, in the factor retention process. However, 22 factors with slight variance differences were excluded prior to further statistical analysis. Illustrated in the scree plot (see Fig. 1), four components stood out in the analysis output. Comparatively, all four factors above the elbow benefited the eigenvalues above 2 with the highest eigenvalue scored as 7.55. The four extracted factors contributed to 21% of the whole variance. Because this contribution was unexpectedly low, we decided to remove items with low factor loadings to optimize the quality of the questionnaire.

**Fig. 2:**
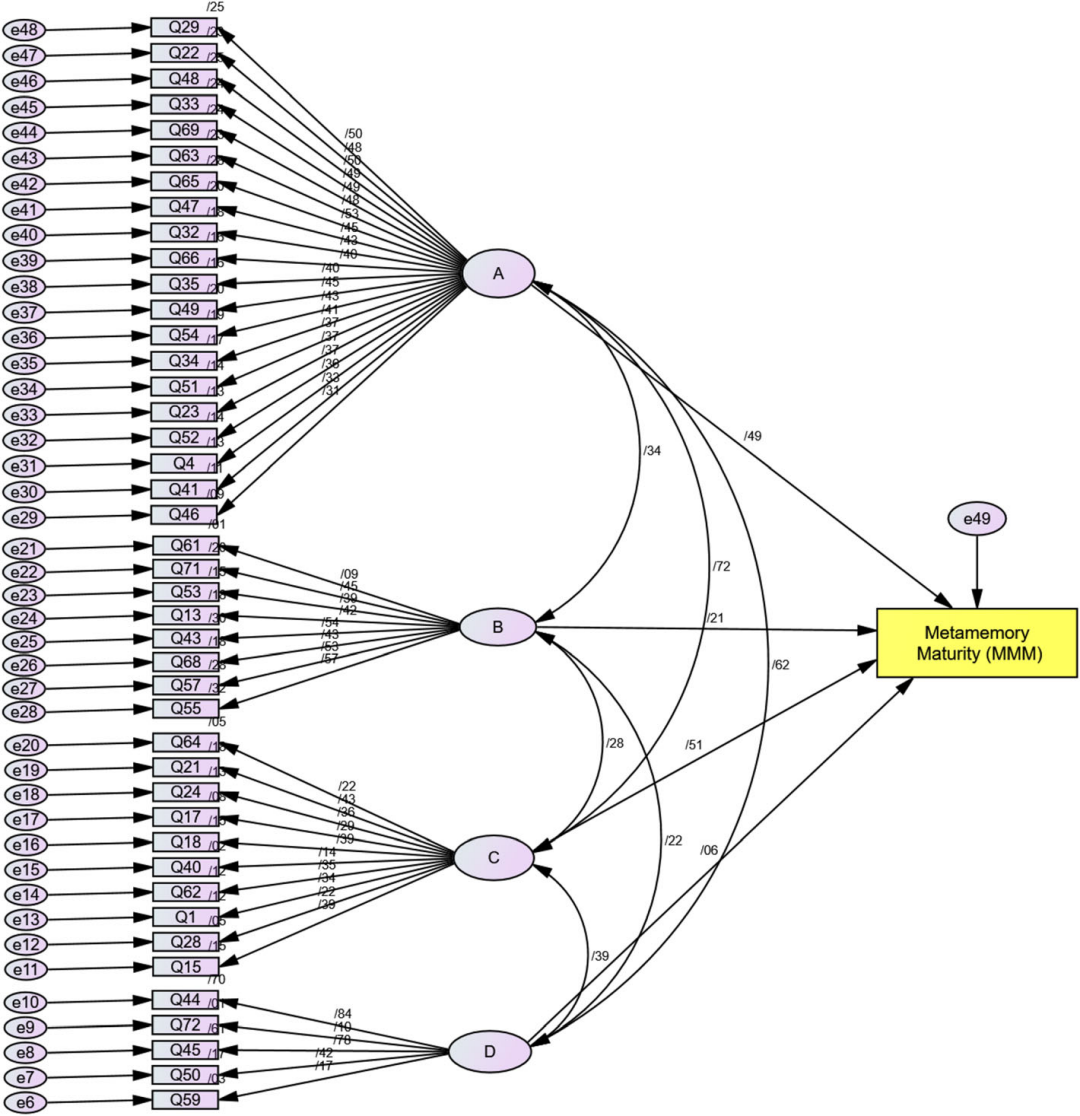
Schematic representation of the first structural model of the MMM questionnaire

In order to detect problematic items, the component matrix was investigated to identify the items that contributed to variations within each component. A few items with cross-loadings were examined, and the items with cross-loadings below 0.20 (Sosik et al., 2009) were removed from the set (*n* = 7). After the second round of content analysis by two professors of applied linguistics, the theoretical framework for the MMM questionnaire was determined by running a structural equation modeling (SEM) in IBM SPSS AMOS 26 (see Fig. 2).

### Construction of the first structural model with 43 remaining items

#### The application of confirmatory factor analysis (CFA)

After eliminating the items with standardized estimates of regression weight below 0.25 (*n* = 18) (Kwan & Chan, 2011), the initial model with four major components included the remaining 43 items in the MMM questionnaire. The surface structure of the model was designed to correspond to the four components in Hultsch et al.’s (1988) model of metamemory. However, for both theoretical and statistical reasons, a confirmatory factor analysis (CFA) was conducted in order to insure the credibility of the model fit.

#### Results of the first structural model’s goodness of fit

The threshold values of RMSEA, GFI, IFI, and TLI were compared to the values in CFA. The measures of chi-square and RMSEA showed significant values (*χ*^2^(405) = 2.123, *p* = 0.00). However, the goodness of fit measures of GFI, IFI, and TLI reported the values of 0.85, 0.81, and 0.80, respectively.

The optimal indices for the goodness of fit have been suggested by several researchers (Browne & Cudeck, 1993; Cho et al., 2020; Kline, 2011). While Browne and Cudeck (1993) recommended the acceptable range of above 0.80 for the goodness of fit (GFI), Cho et al. (2020) and Kline (2011) agreed on GFI greater than 0.90. Hence, the measures of goodness of fit in this study were interpreted as mediocre. In order to increase the credibility of the developed structural model, we excluded some more statistically unfitting items, using their factor loading.

**Fig. 3.**
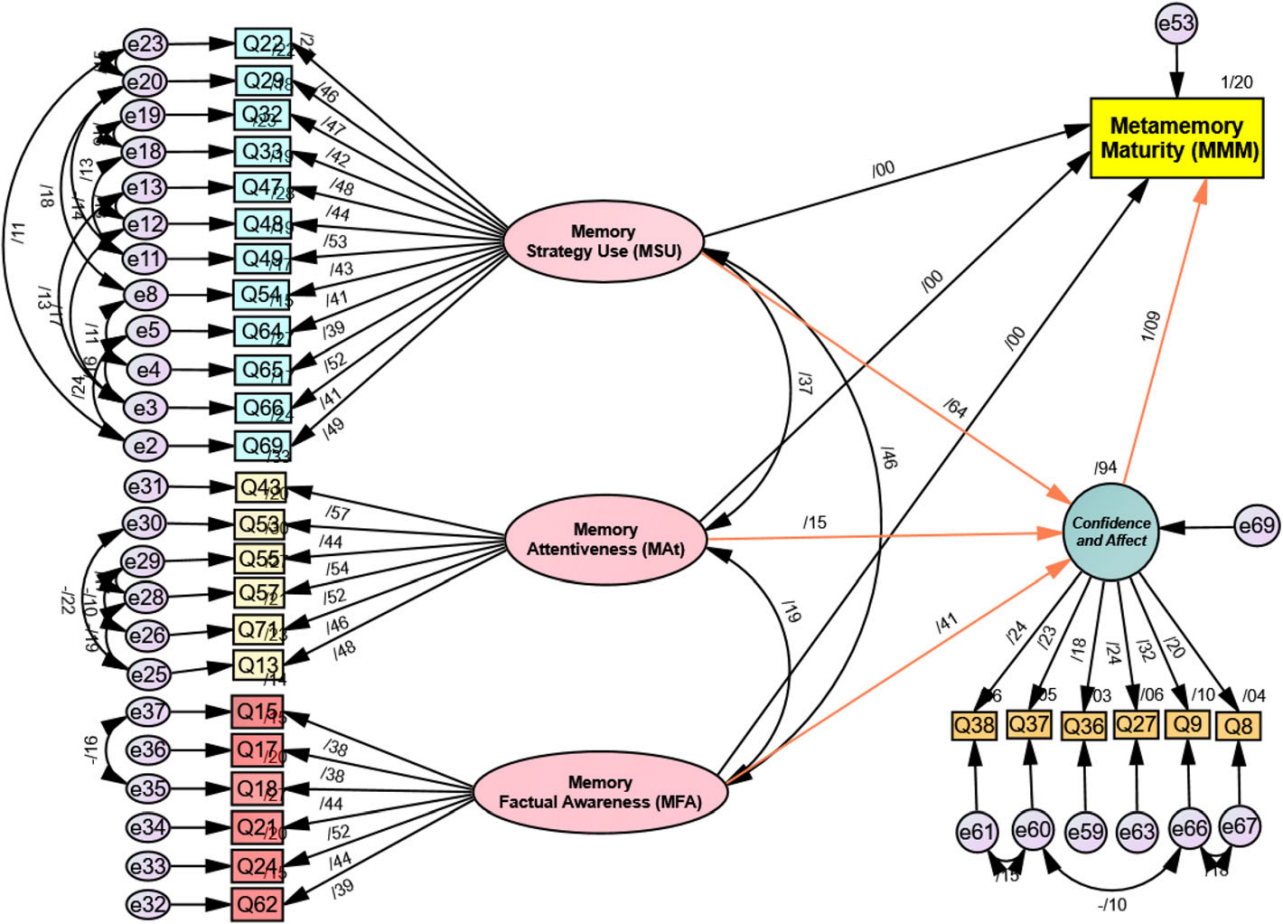
Schematic representation of the final model of the MMM questionnaire

Despite achieving a mediocre GFI for the first developed structural model (Fig. 2), an attempt was made to reconstruct the model. The rationale was to detect more suitable underlying components and path algorithms of metamemory maturity and to plot a model with higher goodness of fit. Further modifications were carried out with a number of items and components by probing through the statistical fits and misfits, so that the second model with different correlational paths and underlying factors was constructed. A notable improvement in the second constructed model was done in terms of re-evaluating the nature of components in the first constructed model, which increased the likelihood of the fourth extracted component to serve as a moderator. Thirteen more items were removed in the final phase of the SEM analysis, which turned the number of items into 30 (see Fig. 3).

### Construction of the second structural model with 30 items

#### Results of the second (finalized) structural model’s goodness of fit

To re-calculate the goodness of fit for the final model of the MMM questionnaire, a reference was made to Hair Jr. et al. (2010). According to their guideline for determining the acceptable factor loadings, for a stereotypical sample size of 350 participants and above, an acceptable factor loading should be set over 0.3 (Hair Jr. et al., 2010). In the final model of the MMM questionnaire, the standardized estimate loadings on every item in the main and moderator components were 0.42 to 0.57, respectively, which were relatively high and acceptable.

The model fit values were calculated by running a chi-square test. The values less than 5 are interpreted as moderate but still acceptable. The values less than 3 are reported as a strong fit. Therefore, the chi-square value (*χ*^2^(407) = 1.434, *p* = 0.00) was interpreted as a desirable fit. RMSEA was reported as 0.035 < 0.05. Other indices of the goodness of fit were also greater than the critical value of 0.90 (IFI = 0.921, GFI = 0.909, CFI = 0.919, and TLI = 0.907). In this round of analysis, therefore, the researchers managed to reach an acceptable measure of goodness of fit (GFI) above 0.9 (see Table 9 in Appendix for the questionnaire items and factor loadings).

#### Path analysis

In addition to factor analysis, a path analysis was conducted to detect the significance of the links across the components and the construct of metamemory maturity in structural equation modeling (SEM). Both direct and indirect paths between the main components, the moderator, and the construct are demonstrated by arrows in Fig. 3. The direct paths among the main components and the construct were labeled as c1, c2, and c3. The indirect paths were shown through the arrows between the main components and the moderator (a1, a2, and a3) as well as the moderator and the construct (b). In the direct and indirect path models, the unrelated paths were programmed to be excluded from the equation to investigate their effects separately. The path construction of the entire model was in accordance with the relevant literature on path analysis and SEM (Kline, 2011).

According to Hair Jr. et al. (2010), all indices related to the moderator in a structural equation model must be significant at *p* < 0.05. The path analysis of the model was conducted by probing the path statistical tables. In order to explore the possible differences between the presence and absence of the moderator, three separate models were designed in IBM SPSS AMOS 26 (i.e., direct, indirect, and moderation models). To ensure the components are significantly connected to the metamemory maturity construct, the direct paths between the main components and the construct were inspected at the outset. The direct contribution of all components was warranted by the significant *p*-values (c1, c2, c3 *p*-values < 0.05). In the direct model, the path coefficients for components 1, 2, and 3 were reported as *β* = 0.70, *β* = 0.17, and *β* = 0.44, respectively.

After the direct path values to the metamemory maturity construct were confirmed, the possibility of indirect path relations was intensified, and the indirect paths were examined. Among the three path values of a1, a2, and a3, only component 1 showed a significant *p*-value (a1 *p*-value = 0.003 < 0.05) with a path coefficient of *β* = 0.64. Components 2 and 3 displayed insignificant paths to the moderator (a2, *β* = 0.15, *p*-value = 0.080 > 0.05, a3 *β* = 0.41, *p*-value = 0.114 > 0.05). Therefore, it was concluded that the moderator only modified the variations in component 1 when it contributed to the metamemory maturity construct. The standardized estimates of the covariance coefficients among the main components were calculated in the next step. The estimates ranged from weak (*σ* = 0.19) between components 2 and 3, relatively moderate (*σ* = 0.37) between components 1 and 2 to a moderate covariance (*σ* = 0.46) between components 1 and 3. The covariance values were evidence of a slight interaction among the components.

The significant interactions in the direct and indirect models set the opportunity for generating the moderation model, which was examined in the final step. In the moderation model, the path coefficients were reported as *β* = 0.70, *β* = 0.17, and *β* = 0.44 for the paths between components 1, 2, and 3 and metamemory maturity, respectively. All three paths were reported to have significant *p*-values (c′1, c′, c′3 < 0.05). Mathieu and Taylor (2006) provided a framework for decisions made on the moderation effect. They suggested that if path c (i.e., direct path from a component to the construct) is reported as significant, the moderation effects should be examined. Then, if the path between the component and the moderator (path a) and the path between the moderator and the construct (path b) are significant, a partial moderation is reported. Eventually, if any of the paths a or b turns insignificant, only the chance of a direct relationship between the components and the construct should be considered. In the final model of the MMM questionnaire, the a1, b, and c′ turned out as acceptable paths; thus, a partial moderation for component 1 was determined. For components 2 and 3, no significant paths to the moderator were decided. Instead, both showed direct paths to the construct (see Fig. 3).

In addition to the coefficients in the path analysis of the final model, the factor loadings of the items that contributed to the main and moderator components were investigated. For the first component, the factor loadings of the 12 items ranged from 0.39 to 0.53. The six items in the second component benefited the factor loadings ranging from 0.44 to 0.57. The third component consisted of six items with factor loadings of 0.38 to 0.52. Finally, the moderator component with six items gained relatively lower factor loadings ranging from 0.18 to 0.32.

#### Validity and composite reliability (CR)

In order to detect the composite reliability (CR) for separate components in the metamemory maturity construct, the standardized regression weights and the correlation values were calculated. As Hair Jr. et al. (2010) noted, the acceptable cutoff point for CR is 0.60 and above. The CR values for components 1, 2, and 3 were all larger than 0.60 (0.798, 0.638, 0.601, respectively). Moreover, to measure the discriminant validity, the researchers examined the average variance extracted (AVE). In a large sample size, the estimation usually results in lower AVE values due to the indicator item loading sensitivity (Hui & Wold, 1982; Lohmöller, 1989). Therefore, the significance of discriminant validity was determined with reference to acceptable measures of CR (above 0.60) obtained in this study. Maximum shared variance (MSV) values were obtained to measure the convergent validity. Except for a subtle violation in component 3, components 1 and 2 benefited the acceptable convergent validity due to a smaller MSV than AVE. In Table 8, the values for the CR, AVE, and MSV are summarized.

**Table 8:** The CR, AVE, and MSV measures

	CR	AVE	MSV	MaxR (H)	Component 1	Component 2	Component 3
Component 1	0.798	0.222	0.208	0.803	0.471	0.367	
Component 2	0.638	0.200	0.135	0.684		0.447	
Component 3	0.601	0.164	0.208	0.618	0.456	0.193	0.405

To sum up, the finalized model of metamemory maturity consists of three main components and a moderator explaining the variations in language learners’ metamemory maturity (see Fig. 3). Component 1, which contained 12 items, was labeled as *memory strategy use (MSU)* as it explores language learners’ active use of memory strategies to memorize items, sort out the items, and manage time in memorization. Component 2 was labeled as *memory attentiveness (MAt)* with six items, which examines how language learners manipulate their attention span to build up strong memories or undivided attention to complex language items. Component 3 was named as *memory factual awareness (MFA)* with six items, which probes into language learners’ overall knowledge of what memory is, how it functions, and how it can be enhanced. The moderator was labeled as *confidence and affect* with six items, which questions the language learners’ level of consciousness, self-control, and engagement.

## Discussion

The present study laid its statistical groundwork to validate the newly developed metamemory maturity (MMM) questionnaire. The first component in the MMM questionnaire, memory strategy use (MSU) includes the largest number of items (*n* = 12). In the literature, ample evidence supports the effective mediation of memory strategy use to the language learners’ memory functionality (Laine et al., 2018; Peng & Fuchs, 2017). Although there are arguments for and against the effectiveness of memory training (e.g., Dunning & Holmes, 2014; Gathercole et al., 2019), the extensive variations in memory span can be easily accounted within the scope of MSU in the MMM questionnaire. In case of training language learners or teachers about how to use memory enhancement strategies such as spaced learning (Nakata, 2015), rehearsal (McKinley & Benjamin, 2020), mnemonics, acronym and associations (Putnam, 2015), and memory palace (Ralby et al., 2017), they will acquire certain skills to plan, employ, and execute effective learning strategies in a variety of language tasks (Klingberg, 2010). In other words, MSU focuses on the reciprocity between metamemory maturity and progressive learning experience.

Memory attentiveness (MAt) as the second component in the MMM questionnaire with six items addresses the language learners’ attention span in the memory-demanding tasks. Several studies support the positive role of language learners’ attentiveness in retaining to-be-memorized items and their long-term retention (Ellah et al., 2019), extensive learning uptake (Small et al., 2020), and successful encoding information with a higher differentiation level (Kilic et al., 2017). In an experimental study, Kilic et al. (2017) reported that in remembering a large number of selective items with similar content, an increased attention span facilitates the language learners’ encoding pathways and processes. Thus, both MSU and MAt are bound to training and constant enhancement on the part of language learners (Wass et al., 2012; Zalbidea & Sanz, 2020).

Third, in the list of the MMM questionnaire components with six items, memory factual awareness (MFA) targets the language learners’ awareness and knowledge about memory system. The knowledge about the functionality of memory system is multifaceted and broad with numerous topics such as types of memory, mechanisms of encoding input, retention and retrieval, and techniques to maintain the brain’s physical health. Empirical studies support how students’ knowledge of memory functionality can initiate self-regulation in their language learning process (Efklides, 2009). Besides, the acute awareness about the negative impacts of factors such as aging or poor diet on memory decrements encourages learners to adopt healthy lifestyle, brain health exercises, and suitable diets to boost brain and memory functionality (Craik et al., 2010). The significant interaction between MSU and MFA in this study (Fig. 3) can be interpreted as the necessity of instructions to the knowledge of memory system, which assists language learners to adopt more efficient memory strategies.

Finally, the moderator role of confidence and affect with six items was explored in the MMM questionnaire. Statistics supported that confidence and affect would regulate the variations in one of the main components of the MMM questionnaire, MSU. The moderator was generated in the final model of the MMM questionnaire for both statistical and theoretical reasons. Regarding the statistics, after items with strong loadings (*n* = 30) defined the main components, the remaining items (*n* = 6) were schematized into a moderating component. Theoretically, confidence and affect were not supported as the moderator component in Hultsch et al.’s (1988) metamemory model; however, “memory-related affect” in their model was an amalgamation of respondents’ emotional and personal attributes. In the MMM questionnaire, language learners’ positive emotions such as self-confidence are assumed to function as a regulator to component 1 (MSU) of metamemory maturity (Margeaux et al., 2017). The role of language learners’ self-confidence in selecting proper memory-related strategy use and spontaneous cognitive offloading is supported in the literature (Auslander et al., 2017; Boldt & Gilbert, 2019).

Despite the structural differences, the MMM questionnaire and the well-known MIA questionnaire (Dixon & Hultsch, 1983) show some similarities in the nature of their components. In MIA, the components of “knowledge of memory processes and tasks” and “cognitive activity” have close theoretical definitions to memory factual awareness (MFA) in the MMM questionnaire, as they all refer to the respondents’ metacognitive awareness. Particularly, in MIA, the component of “frequency of memory strategy use” mirrors that of memory strategy use (MSU) in the MMM questionnaire as they both emphasize the role of acquiring memory strategies. In addition, “perceptions of change in memory capacity over time” in MIA is partially defined as MSU in the MMM questionnaire, both supporting self-monitoring in the respondents. “Locus of control” as another component of MIA also corresponds to memory attentiveness (MAt) in the MMM questionnaire, as both require learners’ ongoing practice of attentiveness. Likewise, the MMM questionnaire and SMSQ questionnaire (Tonković & Vranić, 2011) have some resemblance. Among the six components in SMSQ, “episodic memory, semantic memory, memory for numbers, and visospatial memory” differentiate memory types which are closely related to MFA in the MMM questionnaire as all emphasize the learners’ knowledge of the memory system and functionality. The other two components of “subjective evaluation” and “reminder and aids” in SMSQ can be embedded in MSU in the MMM questionnaire since all address the learners’ active use of memory strategies.

## Conclusion

The metamemory Maturity (MMM) questionnaire was developed and validated in order to explore and evaluate the multi-faceted nature of metamemory maturity in performance on memory-demanding tasks in EFL contexts. The researchers’ major argument in this study is that there is no such concept as weak memory, but an *untrained* memory. This premise was supported statistically, using three analytical techniques of EFA, CFA, and SEM. The developed MMM questionnaire was intended to address the EFL teachers and student teachers in their attempts on memory-demanding tasks such as learning and retaining complex grammatical structures, huge body of new lexical items, or taking turns in an effective verbal communication.

Administering the MMM questionnaire as a placement instrument in educational environments can set an opportunity to analyze and meet the needs of students for receiving instructions to metamemory strategies or engaging in active memory strategy use. In L2 learning and teaching contexts in particular, administering the MMM questionnaire launches a variety of metamemory enhancement strategies by informing teacher trainers about student teachers’ strengths and weaknesses. Using such strategies as verbal and written rehearsals, visual prompts, or mnemonic rhymes, student teachers will intake the required materials for teaching more effectively (Baddeley et al., 2015). This is, in fact, carried out to assist language learners in acquiring such memory-demanding tasks as the sound-letter system of the L2, focusing on language form(s) and expanding the growing body of lexical knowledge.

In terms of the limitations of this study, the following points are in order. First, it should be noted that all the participants were non-native speakers of English whose responses to the questionnaire could be superseded by their sociocultural and first language background (Chun, 2014; Wang & Lin, 2013). In addition, the sample size in the present study did not reach the minimum number recommended in the literature of the SEM studies, so the findings in this study should be interpreted with caution in similar EFL learning contexts. Finally, due to the time limitations and inaccessibility to a larger number of participants at different time intervals, we collected one data set for validation purposes in this study. Ideally, as one of the anonymous reviewers rightly asserted, several rounds of data collection need to be carried out to revise and validate an instrument.

## Data Availability

Please contact the authors for data requests.

## Appendix

**Table 9:** Model fit analysis for 30 items in the final MMM questionnaire

Component	Item number	Item content	Factor loading
Memory strategy use (MSU)	22	I closely observe my memorization progress.	.46
	29	I test my memory regularly right after memorizing items.	.47
	32	Before I memorize items, I think of how I am going to use them.	.42
	33	I memorize new items based on the knowledge, goals, and abilities I already have.	.48
	47	Before I start memorizing items, I focus on my personal motivation.	.44
	48	I observe my feelings during memorization.	.53
	49	When I am memorizing items, I remove myself from any distractors.	.43
	54	I believe when I verbalize my emotions, I can memorize better.	.41
	64	I believe I can memorize a list of 20 items in a very short time.	.39
	65	If I believe in my memory, I can memorize better.	.52
	66	I know a variety of strategies to memorize items.	.41
	69	I regularly challenge myself with memorizing difficult items.	.49
Memory attentiveness (MAt)	57	When I am memorizing items, I will lose my concentration if I have time limit.	.57
	53	When I am memorizing items, I will lose my concentration if I imagine the exam conditions.	.44
	55	The more emotional I am about the items, the better I can memorize them.	.54
	43	Following a cholesterol-free diet makes me recall better.	.52
	71	My negative feelings deteriorate my memorization.	.46
	13	I borrow the strategies of my successful peers for memorization.	.48
Memory factual awareness (MFA)	62	Being healthy or not, I can memorize items equally.	.38
	17	I think humans are born with good or bad memory.	.38
	18	I believe my memories do not change over time.	.44
	21	To memorize better, I look for links between the items.	.52
	24	When I am memorizing, I verbalize the items.	.44
	15	I take notes while I am memorizing.	.39
Confidence and affect	8	When I remember an event, I can recall what exactly happened.	.20
	9	My past memories have no effect on the way I picture my future.	.32
	27	I spend an equal amount of time on memorizing every new item.	.24
	36	I recall a memory separate from my thoughts and imaginations.	.18
	37	Memorizing more items makes me lose some details about items.	.23
	38	It is more difficult to recall the items that are not semantically related to each other.	.24

Goodness of fit (GFI) = .909; comparative fit index (CFI) = .919; Incremental Fit Index (IFI) = .921; Tucker–Lewis index (TLI) = .907; root mean square error of approximation (RMSEA) = .035; *χ*^2^(407) = 1.43

## Abbreviations

MMM: Metamemory maturity
EFL: English as a Foreign Language
MMSSTI: Metamemory, Memory Strategy, and Study Technique Inventory
EMQ: Everyday Memory Questionnaire
SMSQ: Self-Evaluation of Memory Systems Questionnaire
SEM: Structural Equation Modeling
EFA: Exploratory Factor Analyses
CR: Composite Reliability
AVE: Average Variance Extracted
